# Feasibility of Using Multiplayer Game-Based Dual-Task Training with Augmented Reality and Personal Health Record on Social Skills and Cognitive Function in Children with Autism

**DOI:** 10.3390/children9091398

**Published:** 2022-09-15

**Authors:** Daekook M. Nekar, HyeYun Kang, Honnang Alao, JaeHo Yu

**Affiliations:** 1 Department of Physical Therapy, Sunmoon University, Asan 31460, Korea; 2 Department of Computer and Electronic Engineering, Sunmoon University, Asan 31460, Korea

**Keywords:** autism, augmented reality, cognition, personal health record, social skills

## Abstract

The purpose of this preliminary study was to evaluate the feasibility of multiplayer game contents with dual-task exercises using augmented reality (AR) and a personal health record (PHR) system for social skills and cognitive function in children with autism. The present study used a single group pretest–posttest study design with fourteen children diagnosed with autism and aged 6–16 years. The intervention consisted of various game contents designed specifically with cognitive and motor tasks, performed for 30 min per session, twice a week, for three weeks. Outcome measures were conducted before and after the intervention and included social skills and cognitive function. A satisfactory survey was conducted post-intervention to assess the usability of the performed games. As result, statistically significant improvements were observed in all subscales of the social skills and cognitive function expected in two subscales of each measured outcome. Parents and children appreciated the overall game program, and no risk of injury and dizziness were mentioned. This preliminary study found that multiplayer game-based dual-task training using AR and PHR was feasible and has a promising efficacy for children with autism. However, there is the need to conduct a randomized control study with a large sample size.

## 1. Introduction

Autism is a neurodevelopmental disability that affects patients in various domains, such as verbal and non-verbal communication, social reciprocity, the initiation of social relationships, and cognitive function [1]. Generally, children with autism present several symptoms, including restricted interest and repetition of behaviors and limited flexibility in daily routine or environmental changes [2]. The above-described characteristics and symptoms of autism lead to the impairment of social interaction and overall social skills, which are the ability to behave according to a specific situation, such as interactions with others. Social skills are a core characteristic describing autism. Children with social skills impairment present limited eye contact and difficulties in using and understanding gestures or facial expressions. Due to the complexity and high demand for social relationships during adolescence and adulthood, social skills impairment usually increases as the child grows [3]. Therefore, maintaining good social skills is important for children’s healthy development, good relationships with family members and friends, and better academic outcomes [4].

Generally, children or adolescents suffering from autism show impairments in various domains of cognitive function, including working memory, attention, cognitive flexibility, cognitive inhibition, and visual perception ability [5,6]. The impairment of cognitive function in children with autism is one of the hallmarks and has a major influence on individuals’ mental and psychological conditions and causes restricted interest and repetitive behaviors [2]. Cognitive function impairment is also reported to be related to impairment in social skills [7]. Therefore, the improvement of cognitive function may lead to an improvement in social skills.

Many intervention methods are being used to improve cognitive function and social skills in children with autism. Among the various intervention methods, authors in a previous study suggested that cognitive training combined with physical activity may be more effective and advantageous [8]. Theill et al. [9] reported higher improvements in cognitive function and greater impacts on daily functioning in individuals with reduced cognitive function. However, the major limitation of cognitive and social training in children with autism remains the engagement and motivation of the children to participate in the intervention program [10,11]. Recently, the use of technology-based interventions for cognitive and social training for individuals with autism has increased, as the access and use of augmented reality (AR)/virtual reality (VR) in the rehabilitation field has become easier [12]. The use of AR or VR provides various types of feedback to enhance motivation and represents a promising approach for children with autism. Most of the interventions based on AR or VR used game content with various accessories, such as tablets/smartphones, desktops/laptops, Kinect motion sensors, VR goggles, and smartglasses [13]. Most of the mentioned systems were used with individualized game approaches. However, studies revealed that group training has many positive effects and is more advantageous compared to the individualized training approach for increasing social skills and cognitive function [14]. Group training provides possibilities for children to perform recently learned social and cognitive skills with others [15]. However, no prior study assessed the use of group training using AR or VR on social skills and cognitive function in children with autism. Moreover, no prior study included a personal health record (PHR) system to allow parents and children to directly monitor the progress of the intervention and enhance motivation. Furthermore, to our knowledge, no study has proposed offline multiplayer game content with dual-task using goal-oriented training that specifically targets social and cognitive ability in children with autism. Thus, it remains unclear whether group training with multiplayer game content, including cognitive and motor tasks using AR and PHR, is feasible and effective for improving social skills and cognitive function in children with autism.

Therefore, the purpose of this preliminary study was to investigate the feasibility of multiplayer game content with dual-task training using augmented reality and a personal health record system on social skills and cognitive function in children with autism. The findings of this study would provide evidence of the feasibility of game-based dual-task training using AR and PHR in social skills and cognitive function in children with autism. Additionally, it would provide further directions for research on the present topic.

## 2. Materials and Methods

### 2.1. Study Design

This study was a preliminary study with a single group pretest-posttest conducted in the Department of Physical Therapy at Sunmoon University, South Korea. Written informed consent was provided by parents/legal guardians of the participants since participants were all minors (under 18 years old). The study was conducted in accordance with the principles of the Declaration of Helsinki and approved by the Institutional Review Board of Sunmoon University on 16 March 2022 (SM-202112-072-1).

### 2.2. Participants

For this preliminary study evaluating the feasibility of the game-based cognitive-motor training program, we recruited a convenience sample of fourteen children between the age of 6 to 16 diagnosed with autism. The participants were recruited from a local social welfare center located in Asan, South Korea. After receiving a brief explanation regarding the purpose and method of the study, those who expressed their desire to participate were contacted and scheduled to provide informed consent. Only children of parents who provided written consent were included in the study. Autism severity was not considered as part of the inclusion criteria. Inclusion criteria: having been diagnosed with autism; the ability to see, hear and understand basic instructions; and the ability to read and understand Korean (the main language used in the game contents). Exclusion criteria: children with genetic conditions (i.e., fragile X syndrome), those who were not able or did not want to follow the instructor’s directives, and those unable to stand unassisted. All the assessments and interventions were performed at the Department of Physical Therapy, Sunmoon University, by an experienced physical therapist. Table 1 below displays the demographic characteristics of the participants and their parents.

### 2.3. Outcome Measurements

In the present study, we evaluated participants’ social skills and cognitive function at baseline (before intervention) and after the three weeks of intervention. Additionally, we conducted a post-intervention survey on the game-based cognitive-motor training using AR and PHR benefits and enjoyment perception at the end of the last session.

#### 2.3.1. Social Skills

The Social Responsive Scale 2nd edition (SRS-2) was used to assess participants’ social skills. It is a well-known and used measurement tool to provide a continuous measure of social ability related to autism and quantify its severity with good reliability and validity [16]. The scale consists of 65 items, which are dived into five subscales (1) Social Awareness, (2) Social Cognition, (3) Social Communication, (4) Social Motivation, and (5) Restricted Interests and Repetitive Behavior. It was designed to be completed by parents/legal guardians, teachers, or individuals who have been in direct contact with the children for at least one month and know them well. In the present study, the SRS-2 was completed by parents/legal guardians and rated on a 4-point Likert scale. The five subscale T scores were used for data analysis.

#### 2.3.2. Cognitive Function

The Computerized cognitive testing program (CoSAS–S) was used to assess different domains of cognitive function. The CoSAS-s is a tablet-based test, which was designed by NetBlue Ltd. for assessing six domains of cognition, including orientation, memory, attention, visual perception, language, and high-level cognition.

For the orientation test, participants were instructed to choose the correct date (year, month, day, day of week) and the present location (school, hospital, welfare center, home, bank, restaurant, police office) from the predefined choices presented on the screen. Regarding memory, a starfish of different colors appeared one after another on the tablet screen at intervals of 500 to 1500 ms then disappeared and participants were instructed to remember and repeat in the same order the sequences of the color by touching the color displayed on the screen. The assessment of attention was performed in a similar way as the memory test. However, for attention, participants were asked to track a moving ball from different locations and indicate its last location. For the visual perception assessment, participants were instructed to identify objects with similar forms and colors from various objects displayed on the screen. The assessment of language was performed by naming different objects (matching with predefined names) and the high-level cognition assessment consisted of calculation. The CoSAS-S included 29 items that took 10–15 min to complete. The score ranges from 0–100 with the higher score indicating a higher cognitive function level.

#### 2.3.3. Post-Intervention Survey

In order to assess the feasibility of the intervention program in terms of satisfaction and a global overview of usability, we designed a post-intervention survey based on the System Usability Scale (SUS) [17]. Considering the condition of the children, we rephrased and shortened the SUS questions to be easily understandable and relevant to the study context. Hence, item number 2 of the SUS “I found the system unnecessarily complex” and number 8 “I found the system very cumbersome to use” were combined and changed to “Were the games hard to play?”. New questions, such as “Was the game fun and interesting?” and “Did you feel very confident using the program?”, were added to assess how motivating the games were for the children. A question such as “Did your child exhibit any frustration with the game” was added to assess how children perceived the games, since individuals with ASD may exhibit frustration or anger when confronted with a new environment. Additionally, the question “Do you think this program helped your child in the real world?” was added to assess how much the skills learned during the game sessions can be transferred to the real world. Both parents and children were asked to complete the survey at the end of the last session with five questions for parents and seven for children. In order to have a clear-cut opinion from the parents about the program, only one of the parents (the one who assisted in most of the experiment sessions) was asked to fill out the survey. A simple “yes” or “no” rating system was used to allow children to express their perceived feelings using the games with the AR and PHR systems.

### 2.4. Intervention Settings

Participants included in this study completed an intervention program using our designed multiplayer game contents with dual tasks (cognitive and motor tasks) twice a week with 2 sets of 15 min/sessions for 3 weeks (6 sessions in total). The system ran on Windows (Windows 10) with a monitor of 1920 × 1080 resolution. We used the Kinect sensor developer kit to analyze participants’ motion during the performance of the exercises. The sensor integrated into the Kinect uses is a universal serial bus plug-and-play device that translates the scene geometry into depth information. The sensor used has a body-tracking SDK, an RGB and IR camera with an effective depth field of view of 75° H × 65° V, and an angle of 70° with an optimal measuring range of 0.5–3.6 m. Participants’ images were generated at a resolution of 640 × 576 at 30 fps. The information collected, such as the result of the exercise, the angle of joints, and the position of joints, were displayed directly on the monitor and saved for further use.

**Figure 1:**
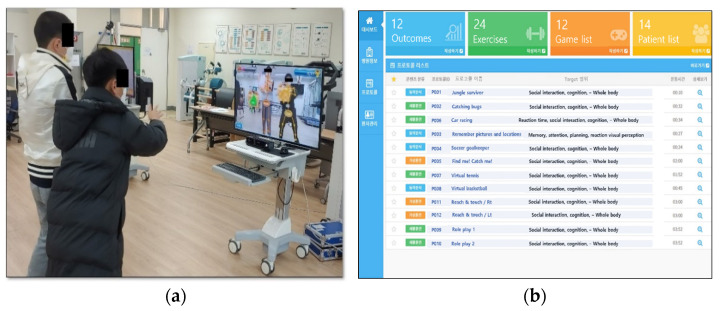
Game-based dual task system. (**a**) Multiplayer game session; (**b**) Manager interface.

We developed multiplayer game contents that specifically included the performance of gross motor movements of the upper extremities, trunk, and lower extremities with the simultaneous performance of cognitive tasks (decision-making, attention, memory, planning, calculation, object color, shape, and size discrimination). The instructions for the game rules were given prior to playing the games through prerecorded video guides and additional real-time audiovisual feedback was provided automatically during the completion of the game through the computer. We included two types of games (cooperative and role-playing games) to allow children to interact with each other in different situations. The cooperative games included contents, such as doubles match tennis with both players on the same side, a 2 vs. 2 basketball with the two players playing as teammates, and a game with the two players working together to catch villains. The role-playing games included games designed to be played by two players, such as one player being a goal striker and the other one a soccer goalkeeper. Participants performed the games in pairs and their respective partners were chosen randomly during the first session (Figure 1a). All participants’ game results were recorded separately and neither player was affected by the other player’s score. The player partners were changed according to their score of the previous session in case one of the players had a lower score of more than 25% compared to their partner. All the participants began the games at the lowest difficulty level and, when mastery was achieved (>95% achievement score), the difficulty was increased gradually in the next session. The game was built with a score ranking system that allows a comparison of the game performance of a single participant between different sessions and a comparison between other participants anonymously. The score ranking system was used to encourage participants to have a goal to achieve and increase self-stimulation. Additionally, based on the evolution of the participants through the games, the system was configurated to suggest game contents with adequate difficulty and speed to enhance furthermore motivation and completion of the games. The system has a user interface showing the game contents and a management interface for therapists and health providers to track the evolution of each participant (Figure 1b). Data saved on the management interface can be securely synchronized to a built-in local cloud server, which was used as the personal health record server. Patients and parents/legal guardians can gain access to the evolution of the intervention process by connecting to the web server with a personalized account using a computer or a mobile phone.

### 2.5. Data Analysis

The IBM SPSS Statistics for Windows version 26.0 (IBM Corp., Armonk, NY, USA) was used for the statistical analysis. Descriptive statistics included the median, mean, range, and standard deviation of all outcome measures. The nonparametric tests were used due to the very small sample size and the parametric assumptions of homogeneity and normality were not met. We conducted the Wilcoxon signed-rank test to compare the pre–post intervention differences and the level of significance was set at *p* < 0.05.

## 3. Results

A total of twenty parents of children diagnosed with autism were briefed about the research study. Sixteen of them presented an interest in the study and booked a time with an investigator to sign the consent and allow their children to participate in the experiments. However, due to the COVID-19 pandemic circumstance and transport issues, two of the sixteen potential participants were enrolled in the experiments. Fourteen children were therefore included in the study and completed the baseline assessment, six sessions of intervention, and the post-intervention assessment. None of the participants withdrew from the study.

The statistical results of the social skills and cognitive function during the comparison of the pretest and posttest are presented below in Table 2 and Table 3. First, the Wilcoxon signed-rank test indicated significant improvements in three subscales (Social Awareness, Social Cognition, and Social Motivation) of the social skills among the five subscales with *p* < 0.05. However, despite no statistically significant result in Social Communication and Restricted Interests and Repetitive Behavior, a decrease in the mean was observed when compared to the baseline data.

Regarding cognitive function, a statistically significant improvement was observed in orientation, memory, attention, and visual perception with *p* < 0.05. However, no improvement was observed in the language and high-level cognition with *p* > 0.05.

Regarding the qualitative analysis of the participants’ and parents’ impressions of the program, all the parents expressed their satisfaction with the overall program, especially for its safety and ease of use. They affirm that playing games with a simple motion capture system without headsets or additional accessories reduces the risk of injury and dizziness. Table 4 below presents the result of the post-intervention satisfactory survey. Parents reported that their children enjoyed the game content and requested the use of the games at home for their children. Only two parents reported that their children presented frustration with the games, whereas twelve of them found the program to be well-integrated and had a benefit on their children in the real world. Thirteen parents found the system to be easy and able to be quickly understood by every child. Only five parents found that the program had some inconsistencies. During the survey, thirteen of the children found the games interesting and expressed their desire to continue playing the games at home. The majority of the children found the system easy to use, felt confident during the play, did not need technical assistance, and were comfortable.

## 4. Discussion

The present preliminary study attempts to determine the feasibility of multiplayer game contents using dual-task exercises (cognitive and motor tasks) with augmented reality and a personal health record system on social skills and cognitive function in children with autism. The main findings were that most of all subscales of the social skills and cognitive function were significantly improved after the performance of the games. The subscales which showed significant improvement were social awareness, social cognition, social motivation, orientation, memory, attention, and visual perception.

The multiplayer game content with dual-task exercises used in the present study required specifically the performance of simultaneous cognitive and motor tasks with whole-body movements. In contrast to previous research, the game protocol in the present study used the interactive cognitive-motor training (ICMT) approach with goal-oriented and role-play contents using motion capture and a personal health record system. The outcomes measurement targeted social interaction ability and cognitive function, which are core problems that children with autism struggle with. To our knowledge, this is one of the first studies that evaluated social skills as well as cognitive function as an outcome measure in a dual-task method (ICMT approach) with AR and PHR for children with autism.

The interaction between the children during the multiplayer game sessions provided the children with an opportunity to experience and develop their ability to work out issues and resolve issues by themselves. The implementation of collaboration between the children appeared to be an opportunity to experiment with challenging social behaviors and develop their critical thinking in a fun manner. It is one of the reasons that explain the improvement in social skills and cognitive function in the present study. Moreover, the performance of simultaneous cognitive and motor tasks known as dual tasks was reported to have a positive impact on cognitive function and memory in patients with various conditions as well as children with developmental disorders [18,19]. The execution of the dual task has a high demand on brain functioning and stimulates the activation of related brain areas. These explanations support the result found in the present study. Moreover, the execution of tasks with a specific goal to achieve generally referred to as a goal-oriented task is important to increase social skills and cognitive function in children with autism. With a specific goal to achieve, participants executed the tasks that lead to the larger, overall accomplishment while continuing to be focused on the desired outcomes. Goal-orientation is reported to positively influence the performance of tasks and enhance motivation [20,21]. Moreover, in our game protocols, we added a ranking system that helped participants execute the tasks by more than their usual capacity in order to surpass their previous score and the peer score as well.

It is important to note that social communication, restricted interests and repetitive behaviors in social skills and language, and high-level cognition in cognitive function did not show significant improvement after the intervention. The game contents did not have an interactive verbal dialogue system to allow the improvement of social communication and language skills. Additionally, we speculated that the improvement of restricted interest and repetitive behaviors, and high-level cognition would require a longer intervention time since changes in the mean score were observed.

In the present study, the easily accessible Kinect camera was used for its motion tracking system and to provide continuous real-time feedback. A personal health record system was built to allow parents and children to directly monitor the intervention process. The data saved on the server was used to adjust the game difficulty level and contents. Despite the nature of this research, which was a preliminary study with a very small sample size, we noticed the feasibility and efficacy of the designed multiplayer game-based cognitive-motor dual-task using AR technology and PHR system for social skills and cognitive function in children with autism.

Additionally, the post-intervention survey provided further information supporting the feasibility and usability of the present program and its protocols. Parents appreciated the program settings and expressed their satisfaction regarding the simplicity and safety of the program. They reported that their children fully enjoyed the games and were positive about using the program at home for continuous training. Moreover, the simplicity of the system does not require a large space and uses the low-cost commercialized motion tracking system Kinect. Children reported that the game difficulty adjustment was adequate and the video instruction for each game was very easy to understand. Additionally, the audio-visual feedback enhances enthusiasm, encouragement, and motivation for participation.

### 4.1. Study Advantages and Implications to Practice

This study presented some advantages and implications for practice. Many studies investigated the use of AR or VR with game content on cognitive function. However, the present study evaluated the feasibility and efficacy of multiplayer games with specific cognitive-motor tasks using augmented reality and a personal health record on cognitive function as well as social skills, which are core problems of children with autism. Moreover, the present preliminary study contrary to other studies used various multiplayer game contents with goal-oriented and role-playing, including cognitive and motor dual-task. Moreover, as a strength of the study design, the outcome measurements were obtained through valid and reliable tools adapted for children with cognitive impairments. The game contents were presented in a random order, reducing the bias between the type of the performed games. The positive feedback provided by both children and parents regarding the game contents and their protocols reinforces the feasibility of the social and cognitive intervention through games in an augmented reality condition for children with autism. The protocols used in the present study can be extended to home rehabilitation sessions to contribute to long-term therapy.

### 4.2. Study Limitations and Future Research Suggestions

Despite all the above strengths, the present study has some limitations to be acknowledged. First, as a preliminary study, the number of participants involved was small (*n* = 14), which limits the generalization of the results and the analysis of the statistical effect size of the game content. Second, the duration of the experiment program was very short (3 weeks, 6 sessions) to assess its long-term effects on social skills and cognition. Third, the absence of a control group with participants playing the same game contents as a single player to assess the effectiveness of the multiplayer content. Therefore, further research should include a control group and conduct a randomized controlled trial with large sample size. Moreover, we could not evaluate the intellectual level of participants, since their parents did not provide consent for the assessment thereof, although the intellectual level of participants should be considered for future research. Aside from providing the *p*-value, further study should provide a quantitative effect size of the multiplayer game on social interaction and cognitive function. It would be interesting to include oral interactive content to target the social communication of children with autism. Moreover, the long-term effects should be studied to evaluate the continuity of the program.

## 5. Conclusions

The present preliminary study aimed to determine the feasibility of multiplayer game content using dual-task exercises (cognitive and motor tasks) with augmented reality and a personal health record system on social skills and cognitive function in children with autism. The study provided evidence that games using dual-task training with augmented reality and a personal health record may be applicable and effective for improving social interactions and the cognitive outcomes of children with autism. The findings showed a promising improvement in social skills and cognitive function, excepting social communication, language, restricted interests and repetitive behaviors, and high-level cognition subscales. However, further studies should be conducted to provide more evidence on the long-term effects of multiplayer gamed-based dual-task training with augmented reality for children with autism.

## Figures and Tables

**Table 1:** Demographic characteristics of participants and parents.

Participants	Total (*n* = 14)
Gender	
Male/Female (number (%))	11/3 (79/21)
Age (years)	12.14 ± 2.24
Height (cm)	162.71 ± 6.17
Weight (kg)	51.79 ± 2.45
Education level (number (%))	
Elementary school	12 (86)
Middle school	2 (14)
**Parents**	**Total (*n* = 14) ^a^**
Gender	
Male/Female (number (%))	4/10 (29/71)
Age (years)	49.17 ± 7.38
Marital status (number (%))	
Married	12 (86)
Divorced or widowed	2 (14)
Employment (number (%))	
Employed	9 (64)
Unemployed	5 (36)

Mean ± standard deviation, ^a^ indicates the number of parents who assisted in the experiment sessions.

**Table 2:** Comparison of social skills before and after the intervention.

Variables	Pre-Intervention	Post-Intervention	Z	*p*
Social Awareness	15.43 ± 1.98	14.79 ± 2.08	−2.165	0.030
Social Cognition	24.07 ± 3.19	23.29 ± 3.14	−2.058	0.040
Social Communication	30.79 ± 4.75	30.14 ± 4.53	−1.725	0.084
Social Motivation	17.50 ± 2.17	16.86 ± 2.68	−2.008	0.045
Restricted Interests and Repetitive Behavior	19.79 ± 2.88	19.29 ± 2.73	−1.706	0.088

*p* < 0.05, mean ± standard deviation.

**Table 3:** Comparison of cognitive function before and after the intervention.

Variables	Pre-Intervention	Post-Intervention	Z	*p*
Orientation	10.57 ± 1.15	11.29 ± 2.09	−1.983	0.047
Memory	11.50 ± 1.91	12.43 ± 1.65	−1.996	0.046
Attention	10.29 ± 1.77	11.21 ± 2.32	−2.032	0.042
Visual perception	10.43 ± 1.34	11.712.36	−1.970	0.049
Language	10.79 ± 1.71	11.50 ± 2.24	−1.876	0.061
High-level cognition	11.43 ± 1.78	12.43 ± 1.82	−1.760	0.078

*p* < 0.05, mean ± standard deviation.

**Table 4:** Post-intervention satisfactory survey.

**Feedback Questions for Children (*n* = 14)**		
Was the game content fun and interesting?	YES	93%
	NO	7%
Do you want to play these games frequently at home?	YES	93%
	NO	7%
Was the program easy to use?	YES	79%
	NO	21%
Did you feel very confident using the program?	YES	71%
	NO	29%
Did you need a technical support to be able to use this game?	YES	7%
	NO	93%
Were you comfortable while playing the games?	YES	86%
	NO	14%
Did the games hard to play?	YES	14%
	NO	86%
**Feedback questions for parents (*n* = 14)**		
Did your child exhibit any frustration with the game?	YES	14%
	NO	86%
Did you find the functions in this program well-integrated for your child?	YES	86%
	NO	14%
Do you think there was too much inconsistency in this program?	YES	36%
	NO	64%
Do you think this program helped your child in the real world?	YES	86%
	NO	14%
Do you think most children would quickly learn to use this program?	YES	93%
	NO	7%

## Data Availability

The data used to support the findings of this study are available from the corresponding author upon reasonable request.
